# Radiation doses during chest examinations using dose modulation techniques in multislice CT scanner

**DOI:** 10.4103/0971-3026.63036

**Published:** 2010-05

**Authors:** Roshan S Livingstone, Joe Pradip, Paul M Dinakran, B Srikanth

**Affiliations:** Department of Radiology, Christian Medical College, Vellore – 632 004, TN, India

**Keywords:** Dose modulation, MSCT, radiation dose

## Abstract

**Objective:**

To evaluate the radiation dose and image quality using a manual protocol and dose modulation techniques in a 6-slice CT scanner.

**Materials and Methods:**

Two hundred and twenty-one patients who underwent contrast-enhanced CT of the chest were included in the study. For the manual protocol settings, constant tube potential (kV) and tube current–time product (mAs) of 140 kV and 120 mAs, respectively, were used. The angular and z-axis dose modulation techniques utilized a constant tube potential of 140 kV; mAs values were automatically selected by the machine. Effective doses were calculated using dose–length product (DLP) values and the image quality was assessed using the signal-to-noise (SNR) ratio values.

**Results:**

Mean effective doses using manual protocol for patients of weights 40–60 kg, 61–80 kg, and 81 kg and above were 8.58 mSv, 8.54 mSv, and 9.07 mSv, respectively. Mean effective doses using z-axis dose modulation for patients of weights 40–60 kg, 61–80 kg, and 81 kg and above were 4.95 mSv, 6.87 mSv, and 10.24 mSv, respectively. The SNR at the region of the liver for patients of body weight of 40–60 kg was 5.1 H, 6.2 H, and 8.8 H for manual, angular, and z-axis dose modulation, respectively.

**Conclusion:**

Dose reduction of up to 15% was achieved using angular dose modulation and of up to 42% using z-axis dose modulation, with acceptable diagnostic image quality compared to the manual protocol.

## Introduction

CT scan has emerged as a powerful tool for effective radiological diagnosis in a variety of diseases since it allows high-resolution three-dimensional images to be acquired in a short period of time. The significant increase in the use of CT scanners has raised concerns about the radiation doses that patients are exposed to and the consequent increase in the probability of occurrence of cancers.[1] The International Commission on Radiological Protection (ICRP) has recommended that patient doses should be kept as low as reasonably achievable (ALARA) consistent with clinical requirements, especially with regard to CT doses.[2] Effective doses generally depend on the individual technique factors as well as the physical size (i.e., weight) of the patients undergoing the CT procedures.[3] One of the easier methods of estimating effective doses is from the dose–length product (DLP) values displayed on the CT console.[4]

The purpose of dose reduction is to increase the benefit/risk ratio of CT, which is turning out to be a major source of exposure to man-made radiation[5] The radiation dose is directly proportional to the tube current at a constant tube potential, scanning time, and slice thickness. Methods of reducing the radiation dose to patients include varying tube current–time product (mAs), increasing the pitch, and reducing the beam energy according to patient body weights.[6] Automatic tube current modulation is one of the tools available in modern CT scanners for radiation dose reduction. This technique adjusts mAs to provide a constant level of image noise on the basis of patient size, attenuation profile, and scanning parameters, including kVp, beam pitch, table speed, and rotation time.[7] While using the dose-modulation technique, mAs can be decreased automatically for regions with lower attenuation whilst maintaining an acceptable level of image noise.[8] The angular-modulation technique involves varying the tube current as the x-ray tube rotates about the patient, while the z-axis modulation involves varying the tube current along the z-axis of the patient.[4] The current study compares the radiation dose and image quality achieved with the use of manual protocol settings with those obtained using the dose-modulation software available in a 6-slice CT scanner, which includes dynamic dose-modulation (D-DOM) and z-axis dose-modulation (Z-DOM) techniques.

## Material and Methods

Two hundred and twenty-one patients who underwent routine CT chest examinations using a 6-slice CT scanner (Brilliance, Philips Medical Systems, Netherlands) were included in the study. The tube potentials available in the machine were 90 kV, 120 kV, and 140 kV. Various other parameters, i.e., the total duration of the scan, field of view, and dose descriptors such as volume CT dose index (CTDI_vol_), DLP, and pitch were displayed on the CT console of the 221 patients, 32 patients were scanned with manual protocol settings (constant tube potential and mAs of 140 kV and 120 mAs, respectively) irrespective of the patients' body weights. These factors were set manually so that an optimal image quality was obtained with the minimum radiation dose. One hundred and ten patients with D-DOM and 79 patients with Z-DOM techniques were scanned with a constant tube potential of 140 kV, with the mAs values automatically selected by the machine. The rotation time was set at 0.75 s, with a pitch of 1.1 and detector collimation of 6 × 3 mm; the reconstructed slice thickness was 5 mm. This study was part of a project funded by the Atomic Energy Regulatory Board (AERB) of India and was carried out after clearance by the ethical committee of the institution where the study was conducted.

All CT examinations were performed as per the standard clinical protocol, with the area scanned extending from the apex of the lungs up to the adrenals. All patients were examined by physicians and were subjected to CT examinations of the chest only when clinically indicated. The patients were categorized into three groups based on their body weight: 40–60 kg (group A), 61–80 kg (group B), and ≥ 81 kg (group C). Patient weights were recorded prior to the CT examinations. All CT chest examinations were performed by injecting contrast agent intravenously, and contrast-enhanced images were acquired using a single breath-hold technique.

The effective doses were estimated by multiplying the DLP values by the normalized coefficient found in the European guidelines on quality criteria of CT, which is 0.017 mSv mGy^−1^ cm^−1^.[9,10] Periodic calibrations using 32 cm polymethyl methacrylate (PMMA) CTDI body phantom with a high-sensitivity 100-mm long pencil ion chamber (CTDI_100_, Victoreen, Ohio, USA) were performed to check the consistency of the CT console values.

Objective evaluation of image quality was based on the measurement of CT image noise of a uniformly attenuating region of the liver. For this purpose, the signal-to-noise ratio (SNR) was measured in a standard 1 cm^2^ circular ROI from the CT console. The ROI selected was one that was not unduly influenced by the contrast in the blood vessels. Two experienced radiologists, blinded to the scanning techniques, independently reviewed each study. The lung, spleen, vessels, adrenals, and esophagus were assessed on a 5-point scale (1 - unacceptable; 2 - substandard; 3 - acceptable; 4 - above average; 5 - superior). All the CT images were assessed with constant window level and a window width of 60 and 400 HU using a high-resolution monitor in the workstation.

## Results

Table 1 shows varying mAs values for D-DOM and Z-DOM for different body weights of patients. Tables 2–4 show CTDI_vol_, DLP, effective dose, and SNR values for patients who underwent CT examinations of the chest. With the use of Z-DOM, the radiation doses were 11% higher than with the manual protocol, without significant variation in the image noise for group C patients. A reduction of 42.3% in radiation dose for group A patients and 20% for patients of group B was observed with the use of Z-DOM compared to the manual protocol. However, there was an increase of SNR by 42% and 29% with the use of Z-DOM for group A and group B patients respectively, compared to the manual protocol. With the use of D-DOM, the tube currents varied from 105–120 mAs, while with Z-DOM, they varied from 27–243 mAs. While using the D-DOM, the tube current-time product (mAs) was adjusted automatically according to patients' weight and did not exceed the preset parameter of 120 mAs.

**Table 1:** Exposure parameters used in manual protocol, D-DOM, and Z-DOM

Weight in kg	Manual settings		D-Dom		Z-Dom	
		
	kV	mAs	kV	mAs (range)	kV	mAs (range)
Group A	140	120	140	114 (109–120)	140	93 (27–180)
Group B	140	120	140	112 (105–118)	140	145 (66–243)
Group C	140	120	140	108 (104–113)	140	182 (119–229)

**Table 2:** CTDI_vol_, DLP, effective doses, and SNR values for manual protocol

Weight in kg	No of cases	CTDI_vol_ (mGy)	DLP (mGy cm) (range)	Mean Effective dose mSv ± SD (range)	Mean SNR (range)
Group A	17	13.7	505 (410–587)	8.58 ± 0.78 (6.97–10)	5.1 (4–6.3)
Group B	12	13.7	502 (403–574)	8.54 ± 0.93 (6.85–9.75)	6.5 (5.4–8.3)
Group C	3	13.7	534 (492–570)	9.07 ± 0.67 (8.36–9.7)	10.3 (7.9–12.9)

SD: Standard deviation, SNR: signal-to-noise ratio

**Table 3:** CTDI_vol_, DLP, effective doses, and SNR values for D-DOM

Weight in kg	No of cases	CTDI_vol_ (mGy) (range)	DLP (mGy × cm) (range)	Mean effective dose mSv ± SD (range)	Mean SNR (range)
Group A	66	13 ± 0.30 (12.4–13.7)	431 (275–514)	7.3 ± 0.54 (4.67–8.7)	6.2 (4.3–11)
Group B	38	12.7 ± 0.42 (11.9–13.5)	446 (389–567)	7.6 ± 0.54 (6.61–9.64)	7 (4–10)
Group C	6	12.4 ± 0.47 (11.8–12.9)	424 (406–445)	7.2 ± 0.28 (6.9–7.6)	9.4 (7.6–12.4)

SD: Standard deviation; SNR: signal-to-noise ratio

**Table 4:** CTDI_vol_, DLP, effective doses, and SNR values for Z-DOM

Weight in kg	No. of cases	CTDI_vol_ (mGy) (range)	DLP (mGy × cm) (range)	Mean effective dose mSv ± SD (range)	Mean SNR (range)
Group A	45	10.6 ± 4.1 (3–20.5)	291.3 (194–430)	4.95 ± 0.97 (3.29–7.3)	8.8 (7–13)
Group B	30	16.5 ± 5.7 (7.2–27.8)	404.2 (287–543)	6.87 ± 1.04 (4.89–9.2)	9.14 (7.3–10.8)
Group C	4	20.8 ± 5.4 (13.6–26)	602 (501–735)	10.24 ± 1.85 (8.5–12.5)	10.6 (9–12.2)

SD: Standard deviation; SNR: signal-to-noise ratio.

Effective doses from D-DOM were found to be consistent, irrespective of the body weights, without significant change in the SNR values. Reduction of radiation dose of up to 21% was achieved with the use of D-DOM compared to the manual protocol. Compared to the use of Z-DOM, a 30% reduction in radiation dose with the use of D-DOM was noted for group C patients. One radiologist reported images from the manual protocol and D-DOM as above average (median = 4) for liver, spleen, adrenals, and esophagus and acceptable (median = 3) for vessels. For Z-DOM, this radiologist reported as acceptable (median = 3) the images of all the organs included in the study. Another radiologist reported images from liver, spleen, adrenals, esophagus, and vessels as acceptable (median = 3) for manual protocol, D-DOM, and Z-DOM.

## Discussion

In diagnostic radiological procedures, the radiation dose and image quality depend on the tube potential and tube current-time product, while in CT scan they depend on tube potential, tube current, scanning time, slice thickness, scanning volume, and pitch. Changes in tube potential affect CT tissue attenuation values and can change tissue contrast in a complex fashion.[11,12] Modern CT scanners have automatic exposure control systems, which permit empirical adjustment of radiological technique factors according to the size of the patient.[13] The use of manual protocol settings would impart the same exposure to all patients, irrespective of the difference in patient sizes If a manual protocol is used, radiation exposures to thin patients can be reduced by selecting lower exposure parameters. In contrast, the use of dose-modulation techniques provides online tube current modulation, so that it is possible to adapt to varying patient sizes without the need for arbitrary selection of exposure values as in manual settings.

A pediatric CT study performed with an online angular-modulation technique showed a 26–43% reduction in the radiation dose compared with manual techniques.[14] Prasad *et al*. reported a dose reduction of 50% by reducing mAs values while maintaining a constant tube potential of 140 kVp.[11] Studies have shown a reduction of 16.9–27% in radiation dose for chest examination by using attenuation-based online tube current modulation.[15,16] As compared with manual settings, a reduction of up to 42% in radiation dose, with acceptable image quality, was noted with the use of Z-DOM in the current study.

It was observed that while using Z-DOM, the radiation dose increased according to the patients' body weights because the mAs values exceeded the preset limit of 120 mAs. There was no significant change in SNR values in Z-DOM, when compared to manual settings, for patients weighing above 80 kg. Both the radiologists reported that the images were of acceptable quality for clinical diagnosis.

In conclusion, a significant reduction in radiation dose to patients, without compromising diagnostic image quality, is possible by using dose-modulation techniques for CT of the chest. The present study reveals that dose reduction of up to 15% can be achieved using D-DOM and up to 42% using Z-DOM, with acceptable image quality. The use of Z-DOM imparted higher doses for patients weighing above 80 kg. Dose modulation software is now available in most modern CT scanners and use of this should be encouraged. The current study suggests that z-axis dose modulation should be used for young adults and for patients of groups A and B and angular dose modulation for group C patients.
